# Decoding the connection: unraveling the role of gut microbiome in fibromyalgia

**DOI:** 10.1097/PR9.0000000000001224

**Published:** 2024-12-24

**Authors:** Amir Minerbi, Arkady Khoutorsky, Yoram Shir

**Affiliations:** a Rambam Health Campus, Haifa, Israel; b Ruth and Bruce Rapaport Faculty of Medicine, Technion Israel Institute of Technology, Haifa, Israel; c Alan Edwards Centre for Research on Pain, McGill University, Montreal, QC, Canada; d Department of Anesthesia, McGill University, Montreal, QC, Canada; e Faculty of Dental Medicine and Oral Health Sciences, McGill University, Montreal, QC, Canada; f Alan Edwards Pain Management Unit, McGill University Health Centre, Montreal, QC, Canada

**Keywords:** Gut microbiome, Chronic pain, Fibromyalgia, Fecal microbiome transplantation

## Abstract

The gut microbiome is emerging as a critical player in the pathophysiology of fibromyalgia, offering mechanistic insights as well as potential diagnostic and therapeutic applications.

## 1. Introduction

Fibromyalgia is a common chronic pain syndrome, characterized by widespread pain, fatigue, sleep disorder, and cognitive fogging.^7,14,19^ Despite its prevalence and impact on the quality of life, the exact etiology of fibromyalgia remains elusive, objective diagnostic tools are lacking, and treatment options are often limited. Recent studies have unveiled a promising avenue of investigation—the role of the gut microbiome in fibromyalgia. The human gut, with its myriads of microorganisms, plays a pivotal role in maintaining the host's health, as well as in variable disorders.^24^ This article explores the intricate relationship between the gut microbiome and fibromyalgia, focusing on emerging research that suggests a potential link between them, and highlighting the implications for future therapeutic interventions.

## 2. The gut microbiome: an ecosystem shaping the host's health

The gut microbiome, a diverse community of microorganisms residing in the gastrointestinal tract, is increasingly recognized as a key player in human health and disease.^24^ Its vast ecosystem not only aids in digestion and nutrient absorption but also exerts profound influences on physiological systems outside the gastrointestinal tract, including the immune, endocrine, cardiovascular, and nervous systems, to name a few. The role of the gut microbiome in various disorders has been increasingly recognized, typically following 4 conceptual research milestones: (1) the establishment of an association between the composition of the gut microbiome and a given medical condition, (2) establishing a causal role for the gut microbiome in the disorder, (3) deciphering the mechanisms allowing the gut microbiome to exert its pathophysiological effects, and (4) harnessing the insights into the role of the gut microbiome in the disorder into diagnostic and therapeutic tools.^31^

## 3. Microbial compositional changes in fibromyalgia

In recent years, several studies have investigated the composition of the gut microbiome in individuals with fibromyalgia.^10,30,32^ Although the overall composition of the gut microbiome in individuals with fibromyalgia is similar to that of healthy controls, specific alterations in the abundance of certain gut bacteria have been observed in patients with fibromyalgia. These included species involved in short-chain fatty acid and bile acid metabolism, as well as species known to modulate host immunity. Bacterial taxa which have been reported to be depleted in patients with fibromyalgia include *Faecalibacterium prausnitzii, Bacteroides uniformis,* and *Prevotella copri.*^10,29,30,32^ These alterations were independent of host and environmental factors such as diet, physical activity, medications, and other medical comorbidities.^28^ Interestingly, despite the high prevalence of irritable bowel syndrome (IBS) among individuals with fibromyalgia,^15^ specific alterations in the composition of the gut microbiome were observed, independent of those associated with IBS.^30^ Furthermore, the relative abundance of certain bacterial taxa was associated with the symptom severity.^30^ These observations provided the first evidence that the composition of the gut microbiome is altered in individuals with fibromyalgia.

## 4. Beyond correlation: the elusive path of causation

Although the associations between the gut microbiome and fibromyalgia are enticing, establishing a causal link between gut microbiome alterations and fibromyalgia is challenging. Do gut microbiome alterations directly contribute to fibromyalgia development, or do preexisting fibromyalgia-related factors indirectly influence the gut microbiome? Recently, the causal role of the gut microbiome was demonstrated in a rodent model.^5^ Gut microbiomes from women with fibromyalgia were transplanted into germ-free mice. Within days following the transplantation, the mice developed pain hypersensitivity, which manifested as spontaneous pain and increased sensitivity to evoked pain. These changes were persistent and did not decay over several months. Inversely, fecal microbiome transplantation (FMT) from healthy individuals did not lead to similar changes in pain sensitization. Moreover, the transplantation of healthy human microbiota reversed pain hypersensitivity in mice that had previously received FMT from humans with fibromyalgia. These experiments demonstrate that the gut microbiome is not only altered in fibromyalgia but that it may play a causal role in the syndrome.

## 5. Mechanisms of gut microbiome's pathophysiological role in fibromyalgia

In recent years, increasing evidence highlights neuroinflammation as an important feature of fibromyalgia. The passive transfer of IgG antibodies from humans with fibromyalgia, but not healthy controls, to mice led to increased sensitivity to noxious mechanical and cold stimuli, and to the loss of intraepithelial nerve fibers.^17^ Small fiber neuropathy is highly prevalent among individuals with fibromyalgia and has been suggested in the pathophysiology of the syndrome.^25,26,34^ Furthermore, the titers of IgG antibodies against satellite glia cells in the plasma of humans with fibromyalgia were associated with symptom severity, with higher titers found in the sera of patients with more severe symptoms.^23^ Cell-mediated immunity has also been implicated in the pathophysiology of fibromyalgia, as the transfusion of neutrophils from humans with fibromyalgia to mice, but not from healthy controls, led to infiltration of neutrophils into the dorsal root ganglia and mechanical hypersensitivity.^6^ The intricate cross-talk between the gut microbiome and the immune system makes it a possible candidate to mediate the effect of the environment on immunity.^37^ Indeed, in our mouse studies, the transplantation of gut microbiota from humans with fibromyalgia led to changes in circulating peripheral mononuclear cells and to microglia activation in the dorsal horn and anterior cingulate cortex in germ-free mice.^5^

Beyond inflammation, the gut microbiome produces a myriad of metabolites that can influence various physiological processes.^22^ In addition to changes in the composition of their gut microbiome, individuals with fibromyalgia show altered serum concentrations of short-chain fatty acids and secondary bile acids,^10,29,30^ some of which may play an important role in nociception. An interesting example is the case of secondary bile acids, which are metabolized by gut bacteria from primary bile acids secreted by the host. These are then absorbed into the circulation, where they exert a multitude of biological activities by binding to peripheral receptors.^18,20^ Furthermore, bile acids are potent immune modulators, owing, in part to bile acid receptors on immune cells.^16^ Bile acid dysregulation has been shown to mediate autoimmune activation in cirrhosis^2^ and multiple sclerosis.^3^ In fibromyalgia, significant changes in bile-metabolizing bacteria are associated with a depletion of certain secondary bile acids,^29^ some of which are known to have an antinociceptive effect.^29^

In conclusion, current evidence suggests that gut microbiota may affect pain hypersensitivity in fibromyalgia both through immune activation and by the disrupted metabolism of bacterial-derived molecules.

## 6. Clinical applications of the gut microbiome in fibromyalgia

Despite the current challenges, exploring the role of the gut microbiome in fibromyalgia holds immense promise for future advancements, including diagnostic and therapeutic applications. The diagnosis of fibromyalgia is currently based solely on medical history, often resulting in delayed or inaccurate diagnoses.^8,13,35^ In this light, using the biological signature of gut bacteria, or their derived metabolites, may hold promise. Based only on the composition of the gut microbiome or bacteria-derived metabolites, machine learning models were able to accurately classify women with fibromyalgia from healthy controls in a cohort of Canadian patients.^29,30^ While these methods require further validation in larger, independent cohorts, identifying specific bacterial signatures associated with fibromyalgia severity and response to different therapies could pave the way for personalized medical approaches. The emerging understanding of the role of the gut microbiome in fibromyalgia opens new avenues for therapeutic interventions.

Nonpharmacological measures, including lifestyle interventions, eg, diet, exercise, regular sleep, and stress reduction, have been shown to modulate the composition of the gut microbiome.^9,11,21,36,37^ Of these, dietary interventions play a cardinal role in shaping the gut microbiome.^1,37^ Several dietary interventions have shown potential in small clinical trials.^4,27,33^ As dietary intake appears to affect the symptom severity, its effects on the composition and function of the gut microbiome may be one putative mechanism. Further research is needed to investigate the effects of diet on bacterial taxa differentially abundant in fibromyalgia, but one could hope that personalized nutrition plans that consider the individual's gut microbiome profile may offer targeted approaches for managing fibromyalgia symptoms in the future.

Fecal microbiome transplantation, a procedure involving the transfer of fecal material from a healthy donor to a recipient, has shown promise in treating certain gastrointestinal disorders associated with altered microbiome compositions. In 2 recent open-label clinical trials, FMT from healthy women led to decreased pain and overall symptomatic burden in individuals with fibromyalgia.^5,12^ Although these preliminary reports must be taken cautiously, they do provide hope that targeting the gut microbiome may allow for novel, mechanism-based treatment modalities for fibromyalgia.

## 7. Conclusion

The intricate interplay between the gut microbiome and fibromyalgia is a burgeoning field of research that holds promise for unraveling the mysteries surrounding this complex disorder. Alterations in the gut microbial community appear to contribute to the immune activation and dysregulated circulating metabolite profile observed in patients with fibromyalgia. Understanding these connections opens new avenues for diagnostic and therapeutic interventions such as dietary modifications and FMT that might emerge as potential strategies to restore balance within the gut microbiome.

Although this research is promising, more studies are needed to confirm the potential benefits of targeting the gut microbiome for treating fibromyalgia. Finally, the potential impact of harnessing the gut microbiome's therapeutic interventions arguably extends beyond fibromyalgia, offering insights into the broader connections between gut health and chronic pain disorders.

## Disclosures

The authors have no conflict of interest to declare.
